# 

**Published:** 1859-05

**Authors:** 


☞ *For Receipts, see 3d Page of Cover.*

### EDITORIAL.

### TO OUR PATRONS.

Our thanks are due to those subscribers, who have so promptly complied with our request to forward the amount of their subscriptions. Their promptness confirms us in the belief that this journal will receive a generous support. To those still in arrears, we respectfully renew the request already made, to forward the amount due.

We have received a number of letters, complaining that money formerly sent has not been credited. We have no other guide, except the books as they were delivered to us, but all moneys sent will be credited as soon as the fact is known to us.

Any persons who have forwarded money by mail, and who are not credited, are respectfully requested to give us early notice.

### RECEIPTS FOR THE JOURNAL UP TO MAY 7, 1859.

### ILLINOIS.

| | | |
|---|---|---|
| Dr. A. S. Bam, | vol. 2, 1859, | $2 |
| " Thos. Hoffman, | " " | 2 |
| " J. S. Lawrence, | " " | 1 |
| " H. D. Mott, | " " | 2 |
| " Thos. Winston, | " " | 2 |
| " E. Smith, | " " | 2 |
| " H. T. Bartlett, | " " | 2 |
| " P. W. Hill, | " " | 2 |
| " J. G. & Z. H. Whitmire, | " " | 2 |
| " John Emery, | " " | 2 |
| " E. R. Boardman, | vols. 1 & 2, 1858-9, | 4 |
| " Jas. H. Fries, | " " " | 4 |
| " J. C. Jewett, | " " " | 4 |
| " Geo. Higgins, | " " " | 4 |
| " Geo. T. Allen, | vols. 1, 2 & 3, 1858-9-60, | 5 |
| " J. C. Patterson, | vol. 1, 1858, | 2 |
| " John Wright, | " " | |

### INDIANA.

| | | |
|---|---|---|
| Drs. C. D. & J. C. Pearson, | vol. 2, 1859, | $2 |
| Dr. L. D. Glazebrook, | " " | 2 |
| " John Dancer, | " " | 2 |
| " Calvin West, | " " | 2 |

### MICHIGAN.

| | | |
|---|---|---|
| Dr. Henry Knapp, | vols. 1 & 2, 1858-9, | $4 |
| " L. W. Lovell, | vol. 2, 1859, | 2 |

### IOWA.

| | | |
|---|---|---|
| Dr. H. Fish, | vol. 2, 1859, | $2 |
| " J. H. Foster, | " " | 2 |
| " F. Heise, | " " | 2 |
| " James Carson, | vol. 1, 1858, | 1 |

### WISCONSIN.

| | | |
|---|---|---|
| Dr. A. B. Cary, | vol. 2, 1859, | $2 |
| " Rowley Morris, | " " | 2 |

### MISSOURI.

| | | |
|---|---|---|
| Dr. J. W. Bird, | vol. 2, 1859, | $2 |
| " Wm. G. Elder, | " " | 2 |

### NEBRASKA.

| | | |
|---|---|---|
| Dr. Wm. Arnold, | vol. 2, 1859, | $2 |

